# Protective effects of a comprehensive topical antioxidant against ozone-induced damage in a reconstructed human skin model

**DOI:** 10.1007/s00403-020-02083-0

**Published:** 2020-05-08

**Authors:** Alessandra Pecorelli, David H. McDaniel, Mitchell Wortzman, Diane B. Nelson

**Affiliations:** 1 NC State University, Plants for Human Health Institute, Kannapolis, NC USA; 2 McDaniel Institute of Anti-Aging Research, Virginia Beach, VA USA; 3 skinbetter science, LLC, 3200 E Camelback Rd, Suite 395, Phoenix, AZ USA

**Keywords:** Antioxidants, Ozone, Oxidative damage, Cosmeceuticals, Free radicals, Reactive Oxygen Species (ROS)

## Abstract

Tropospheric ozone (O_3_) is a source of oxidative stress. This study examined the ability of a topical antioxidant (WEL-DS) to inhibit O_3_-mediated damage in a human epidermal skin model. Four groups of tissues (*N* = 24) were compared: Group 1 (control) were untreated and unexposed; Group 2 were untreated and exposed to O_3_ (0.4 ppm, 4 h); Group 3 were pretreated with WEL-DS and unexposed; Group 4 were pretreated with WEL-DS and exposed to O_3_ (0.4 ppm, 4 h). Pretreated tissues were topically treated with 20 uL of WEL-DS and incubated for up to 20 h at 37 °C [humidified, 5% carbon dioxide (CO_2_)]. After 24 h, tissues were re-treated with WEL-DS and exposed to O_3._ Tissues were evaluated for Reactive Oxygen Species (ROS), hydrogen peroxide (H_2_O_2_), 4-hydroxynonenal (4-HNE) protein adducts, NF-κB p65 response and histology. In O_3_-exposed groups, WEL-DS significantly inhibited ROS formation vs. untreated tissues (*p* < 0.05). Pretreatment with WEL-DS inhibited H_2_O_2_ production vs. untreated tissues (*p* < 0.05), and decreased NF-κB p65 transcription factor signal. Oxidative stress induction in O_3_-exposed tissues was confirmed by increased levels of 4-HNE protein adducts (marker of lipid peroxidation); WEL-DS application reduced this effect. WEL-DS inhibited damage in tissues exposed to O_3_ with no significant changes in epidermal structure. A comprehensive topical antioxidant significantly diminished O_3_-induced oxidative damage in a human epidermal skin model.

## Introduction

Skin provides a protective barrier against radiation from ultraviolet (UV) exposure and other external environmental stressors, and has a natural ability to protect itself against the effects of aging, harmful UV radiation, and exposure to other environmental factors such as ozone (O_3_) through an elaborate antioxidant defense system [1–9]. These external environmental stressors generate free radicals that, coupled with intrinsic aging, can overwhelm the skin’s natural endogenous defenses, causing oxidative damage [1, 2, 8–10]. As skin loses its ability to function efficiently, this leads to suboptimal protection against infection, cancer, as well as accelerated skin aging [1, 2, 8, 9, 11, 12].

Tropospheric O_3_ is produced by chemical reactions between oxygen and air pollutants in the presence of sunlight. Lungs and skin are the main target organs directly exposed to tropospheric O_3_. It does not penetrate the deeper layers of skin, but acts via oxidative stress and inflammation [13]. Ozone is not a radical species per se; rather, it is a highly reactive pollutant that depletes endogenous antioxidants, triggering a cellular response and generating free radicals that lead to lipid peroxidation [13–15]. Among the by-products of lipid peroxidation are the alpha–beta unsaturated aldehyde 4-hydroxynonenal (4-HNE) and isoprostanes like 8-iso-prostaglandin-f(2α) [13, 16]. 4-HNE is a recognized marker of oxidative stress and important in mediating various signaling pathways [16]. In addition, oxidative stress from O_3_ can activate redox-sensitive transcription factors, particularly NF-κB [13], triggering inflammatory responses. Furthermore, O_3_ has been shown to influence the activity of enzymes involved in the turnover and degradation of connective tissue, such as expression of metalloproteinase (MMP)-9 [13]. These molecular mechanisms induced by a cumulative exposure to O_3_ lead to tissue damage and compromise skin barrier integrity.

Sunscreens protect skin against damage caused by exposure to UVA and UVB radiation by absorbing, blocking and scattering UV, but mechanistically do not have the ability to protect against damage caused by free radicals. Through the generation of free radicals and Reactive Oxygen Species (ROS), O_3_ depletes antioxidant levels in the skin. Topical antioxidants penetrate the skin to neutralize free radicals, preventing damage to cells [17–19]. Thus, topical antioxidants can potentially protect against O_3_-induced skin damage and enhance skin’s natural antioxidant defenses [19–24]. As such, topical antioxidants act as an additional safety net to sunscreens [17, 19, 23].

Topical antioxidants provide unique properties and benefits in combating free radicals depending upon their source. Hydrophilic (water-soluble) antioxidants protect the water-containing portions of cells, interior cell structures, and interstitial fluid [20, 21]. Enzymatic antioxidants such as superoxide dismutase and ubiquinone support the body’s internal defenses and protect mitochondria. Lipid-rich components of cells, including cell membranes, are protected by hydrophobic or lipid-soluble antioxidants, such as vitamin E [20, 21]. Developing an effective multi-sourced topical antioxidant is challenging owing to the inherent chemical instability of antioxidants [22].

Comprehensive skin protection requires a topical product that combines various antioxidants to facilitate synergistic interaction and provide broad-based protection from various types of ROS across all cellular levels of the skin [19, 25, 26]. Developed utilizing advanced formulating chemistry techniques, WEL-DS, a comprehensive topical antioxidant, combines a balanced ratio of 19 potent water-soluble, enzymatic, and lipid-soluble antioxidants, designed to provide broad-range protection of the skin from free radical damage (Table 1). Prior studies have demonstrated WEL-DS’s ability to quench free radicals, protect skin from the oxidizing effects of UV radiation, and visibly reduce the effects of facial photodamage [27].

**Table 1:** WEL-DS antioxidant ingredients

Water-soluble antioxidants	Enzymatic antioxidants	Lipid-soluble antioxidants
Chlorogenic acids	*Arabidopsis thaliana* extract	Tetrahexyldecyl ascorbate (vitamin C)
*Coffea arabica* leaf extract	Superoxide Dismutase (SOD)^a^	Tocopheryl acetate/tocopherol (vitamin E)
*Theobroma cacao* seed extract (cocoa)	Ubiquinone (CoEnzyme Q10)^b^	*Glycyrrhiza glabra* root extract (licorice)
Ergothioneine		Ubiquinone (CoEnzyme Q10)^b^
*Curcuma longa* root extract (turmeric)		
*Euterpe oleracea* fruit extract (acai)		
*Vitis vinifera* seed extract (grape seed)		
*Buddleja officinalis* flower extract		
*Camellia sinensis* leaf extract (green tea)		
Carnosine		
*Crocus sativus* leaf extract (saffron)		
*Olea europaea* fruit extract (olive)		
Superoxide dismutase (SOD)^a^		

^a^Antioxidants that are both water-soluble and enzymatic

^b^Antioxidants that are both lipid-soluble and enzymatic

The study described herein evaluated the ability of WEL-DS to protect skin against O_3_-mediated damage utilizing a reconstructed human epidermal skin model.

## Materials and methods

### Reconstructed human epidermal skin model and experimental design

Reconstructed human epidermal skin tissues (EpiDerm™ model, MatTek Corporation, Ashland MA) were incubated at 37 °C and 5% CO_2_ overnight. Tissues were then divided into four groups, each containing six tissue samples (*N* = 24): Group 1 tissues served as an untreated, unexposed control; Group 2 tissues were untreated and exposed to 0.4 parts per million (ppm) of O_3_ for 4 h; Group 3 tissues were pretreated with WEL-DS and unexposed, and Group 4 tissues were pretreated with WEL-DS and exposed to 0.4 ppm of O_3_ for 4 h.

### WEL-DS pretreatment and O_3_ exposure

Pretreated tissues in Groups 3 and 4 were topically treated with 20 µL of WEL-DS and incubated for up to 20 h at 37 °C in a humidified 5% CO_2_ atmosphere. Following overnight treatment, the tissues were treated with a second application of WEL-DS and exposed to 0.4 ppm of O_3_ (Groups 2 and 4) or filtered air (Groups 1 and 3) for 4 h.

Ozone was generated from O_2_ by an electrical corona arc discharge generator (Model 306 ozone Calibration Source, 2B Technologies, Ozone Solution USA). The O_2_–O_3_ mixture (95% O_2_, 5% O_3_) was combined with ambient air and introduced into a Teflon-lined exposure chamber, continuously ventilated. O_3_ concentration in the chamber was adjusted to 0.4 ppm at the input of the circuit and continuously monitored by an O_3_ detector at the output port of the circuit. Exposure to filtered air was carried out in a similar exposure chamber. Temperature and humidity were monitored during exposures.

### Measurement of intracellular ROS levels

After the second application of WEL-DS and before O_3_ exposure, a part of tissues (*N* = 3 per condition) were incubated in the dark with 340 μM 2′,7′-dichlorofluorescein diacetate (DCF-DA) in DPBS for 30 min at 37 °C in a humidified 5% CO_2_ atmosphere, to determine intracellular ROS levels. Then, tissues were rinsed with DPBS, placed in a new plate with culture medium and exposed to O_3_ 0.4 ppm for 4 h. At the end of O_3_ exposure, DCF fluorescence, a measure of ROS production, was determined by SpectraMax iD3 plate reader (Molecular Devices, LLC., San Jose, CA, USA), at 485 nm (excitation filter) and 530 nm (emission filter).

### Hydrogen peroxide (H_2_O_2_) production

The levels of H_2_O_2_ in the media were evaluated by the Amplex Red-Horseradish Peroxidase (HRP) method. Amplex Red reacts with H_2_O_2_ in an HRP-catalyzed reaction, producing highly fluorescent resorufin. Briefly, at the end of the O_3_ exposure (4 h), 10 μl of maintenance medium from each tissue was added to a 96-well plate and, then, a working solution containing 25 µM Amplex Red reagent and 0.5 U/ml HRP were added to each well. After 30 min incubation in the dark, fluorescence was detected with a Synergy H1 Hybrid Multi-Mode Reader (BioTek Instruments, Inc., Winooski, VT, US) with excitation/emission wavelengths of 530 nm/590 nm. H_2_O_2_ accumulation in the medium was calculated by comparing its fluorescence with that of an H_2_O_2_ standard curve. H_2_O_2_ production was expressed in micromolar units.

### 4-Hydroxynonenal (4-HNE) protein adducts levels

Following O_3_ exposure, tissues (*N* = 3 per condition) were placed at 37 °C in the 5% CO_2_ incubator overnight. After 24 h, tissues were rinsed several times with DPBS and then incubated with 0.02% trypsin/EDTA for 2 min at 37 °C, and washed again with DPBS. Then, tissues were snap-frozen in liquid nitrogen and homogenized in a lysis T-PER buffer containing 1% protease inhibitors and 1% phosphatase inhibitors. After centrifugation at 17,115*g* at 4 °C, the supernatants were collected and protein concentration was measured by Bradford assay (Bio-Rad Laboratories, Inc., Hercules, CA, USA). Levels of 4-HNE protein adduct in tissue lysates were measured using an ELISA kit (OxiSelect™ HNE-His Adduct ELISA kit, Cell Biolabs, Inc., San Diego, CA), according to the manufacturer’s instructions. The content of 4-HNE protein adducts was spectrophotometrically evaluated at 450 nm and expressed in µg/mg protein.

### Histological analysis and immunofluorescence

General morphological structure of different epidermal layers in tissues were analyzed using hematoxylin and eosin (H&E) staining. In addition, the NF-κB pathway was evaluated by immunofluorescence for p65 subunit. As previously described, following O_3_ exposure, tissues (*N* = 3 per condition) were placed at 37 °C in the 5% CO_2_ incubator overnight. After 24 h, tissues were fixed by immersion in 10% neutral-buffered formalin (NBF) at RT and then paraffin embedded. Sections (4 μm) were deparaffinized in xylene and rehydrated in alcohol gradients, before H&E staining or immunofluorescence analysis. For immunofluorescence, after a heat-mediated antigen retrieval step in pH 6.0 citrate buffer, tissues were blocked in 5% normal goat serum for 1 h and, then, incubated with NF-κB p65 antibody overnight at 4 °C. Slides were then incubated with a secondary fluorescent goat anti-rabbit Alexa Fluor 488 antibody for 1 h at RT in the dark. The slides were mounted with ProLong Gold Antifade Mountant with 4′, 6-diamidino-2-phenylindole (DAPI) for nuclei staining. Negative control sections were processed by omitting primary antibody. Images were acquired using a Zeiss Z1 AxioObserver LSM10 confocal microscope equipped at 40 × magnification.

### Statistical analysis

Statistical analyses were performed using GraphPad Prism software (GraphPad Software, La Jolla, CA). Results were expressed as mean value ± SD. Data among multiple groups were compared using two-way ANOVA, followed by Tukey’s post hoc test for multiple comparisons. Differences were considered statistically significant if *p* < 0.05, *p* < 0.01, or *p* < 0.001.

## Results

### Intracellular ROS production

**Fig. 1:**
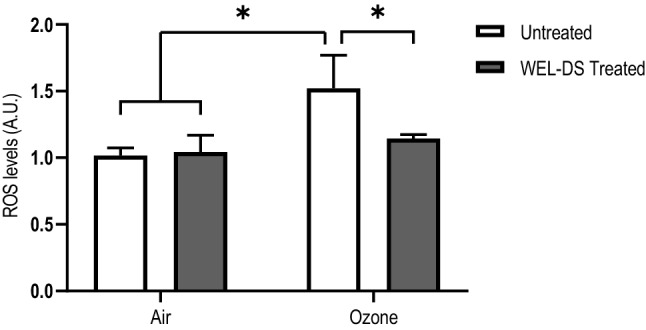
Pretreatment with WEL-DS inhibited ROS production in O_3_-exposed tissues (*p < 0.05)

As determined by DCF-DA assay, exposure to 0.4 ppm of O_3_ induced a significant increase of ROS production in untreated exposed tissues compared to air-exposed tissues (*p* < 0.05; Fig. 1). Application of WEL-DS significantly reduced ROS formation in pretreated tissues exposed to O_3_ compared with tissues that were untreated and exposed to O_3_ (*p* < 0.05; Fig. 1).

### H_2_O_2_ formation

**Fig. 2:**
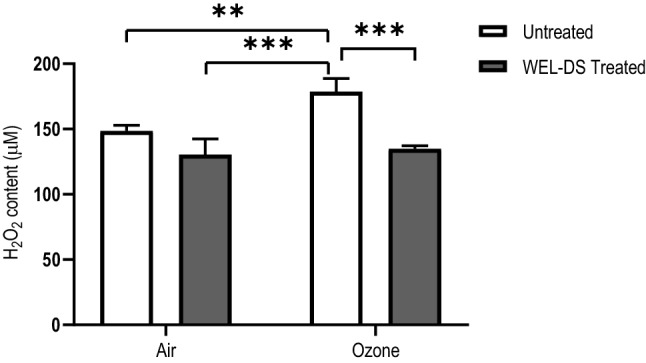
Pretreatment with WEL-DS inhibited H_2_O_2_ formation in O_3_-exposed tissues (**p < 0.01 and ***p < 0.001)

A specific and commonly known ROS, H_2_O_2_, was evaluated. As seen in Fig. 2, there was a significant increase of H_2_O_2_ after 4 h of O_3_ exposure in untreated tissues compared to air-exposed tissues (*p* < 0.01 and *p* < 0.001), significantly different from untreated and WEL-DS-treated tissues, respectively. In O_3_-exposed tissues, topical pretreatment with WEL-DS decreased H_2_O_2_ production compared to untreated tissues (*p* < 0.001; Fig. 2).

### 4-HNE protein adducts levels

**Fig. 3:**
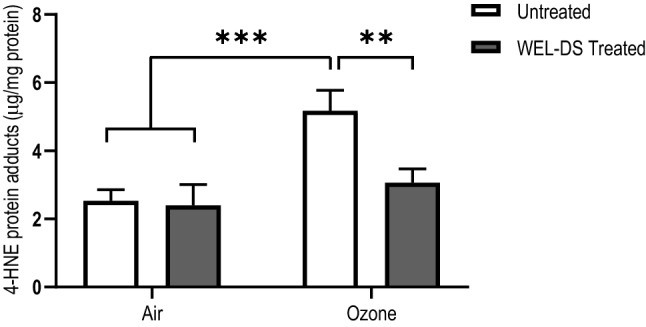
WEL-DS reduced the formation of 4-HNE protein adducts in O_3_-exposed tissues (**p < 0.01 and ***p < 0.001)

Evidence of induction of oxidative stress in tissues exposed to O_3_ was confirmed by increased levels of 4-HNE protein adducts, a marker of lipid peroxidation and protein oxidative damage. Twenty hours following O_3_ exposure, there was a significant increase in 4-HNE protein adducts in untreated exposed tissues compared to air-exposed tissues (*p* < 0.001; Fig. 3). In O_3_-exposed tissues, pretreatment with WEL-DS significantly reduced this effect compared to untreated, O_3_-exposed tissues (*p* < 0.01).

### NF-κB p65 protein expression

**Fig. 4:**
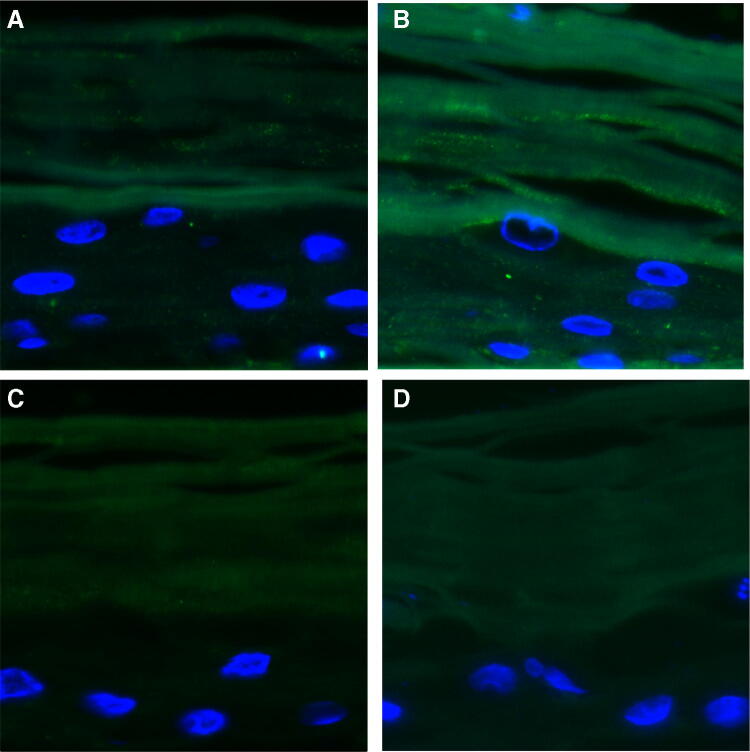
Pretreatment with WEL-DS prevented the increase in NF-κB protein expression (green fluorescence), following exposure to O_3_. **a** Air. **b** O_3_ exposure. **c** WEL-DS. **d** WEL-DS + O_3_ exposure. Nuclei (blue) were stained with DAPI. Original magnification ×40

The ability of O_3_ exposure to induce inflammatory signaling pathways in the skin is well known [13]. To test the effect of WEL-DS to inhibit inflammatory responses triggered by O_3_, NF-κB p65 expression was evaluated in tissues by immunofluorescence. As indicated by the enhanced green fluorescence signal in Fig. 4b, there was an increase in NF-κB p65 protein in skin tissues exposed to O_3_. Of note, pretreatment with WEL-DS was able to prevent this effect, as noticed by the lower intensity of NF-κB p65 green signal in tissues pretreated and exposed to O_3_ (Fig. 4d). No difference in NF-κB p65 protein expression was observed in control tissues (Fig. 4a, c).

### Histopathologic examination

**Fig. 5:**
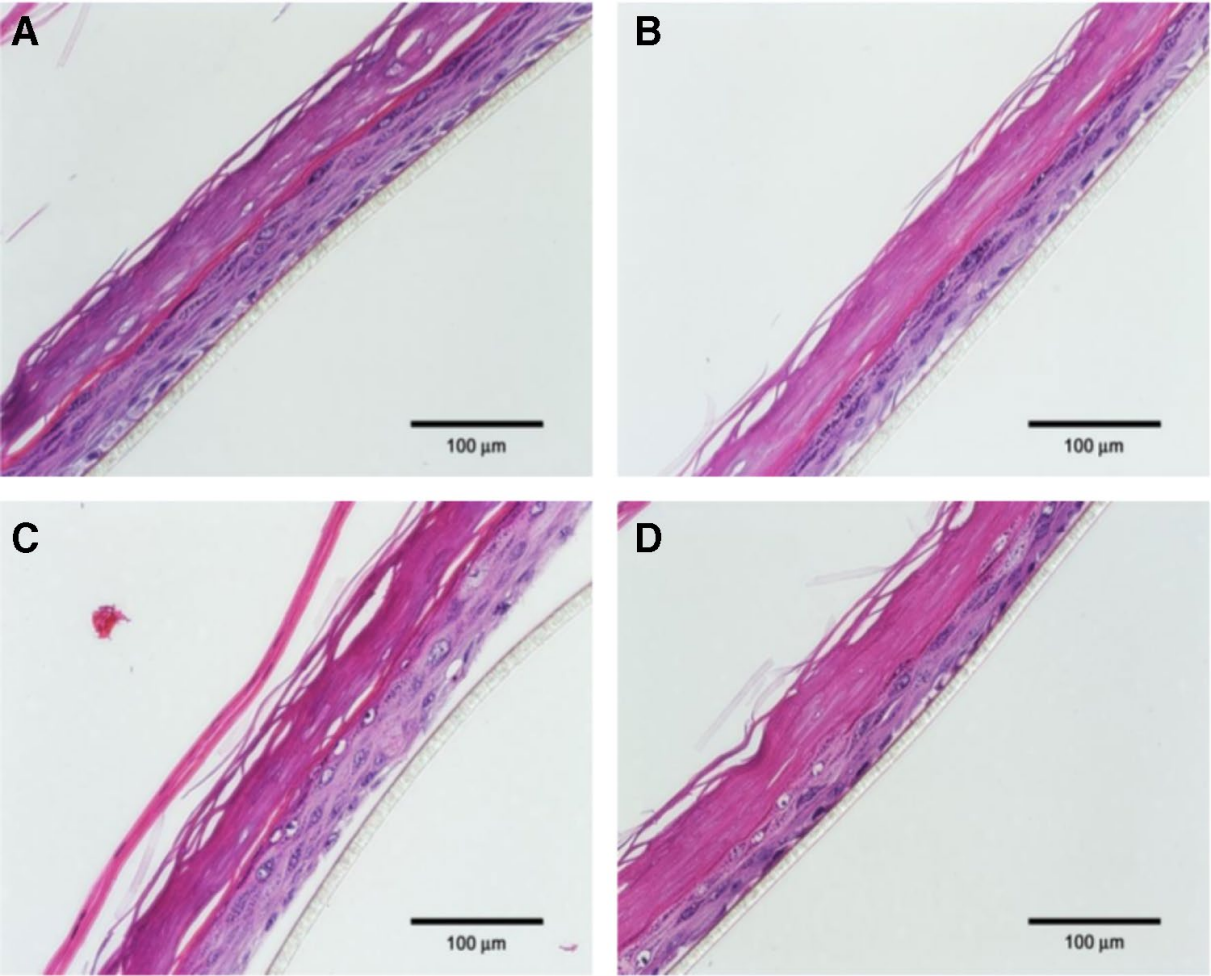
Pretreatment with WEL-DS inhibited structural damage in O_3_-exposed tissues. **a** Normal control—no O_3_ exposure. **b** WEL-DS—no O_3_ exposure. **c** Untreated + O_3_ exposure. **d** WEL-DS + O_3_ exposure

Histopathological examination demonstrated the protective effects of WEL-DS on epidermal tissues exposed to O_3_ (Fig. 5a–d). Morphological alterations were observed in untreated tissues exposed to O_3_ with tissues appearing separated and disorganized (Fig. 5c). Conversely, WEL-DS inhibited tissue damage in pretreated tissues exposed to O_3_ with no significant changes in epidermal structure with the epidermis remaining intact and compact in appearance, similar to that of the unexposed control (Fig. 5a, d, respectively).

## Discussion

Tropospheric O_3_ is one of the most toxic environmental stressors to skin. Research using an in vivo skin model demonstrated an increase of proliferative, adaptive and pro-inflammatory cutaneous tissue responses to O_3_ exposure [28]. Upon interaction with the lipid-rich plasma membrane of the skin barrier, O_3_ exposure generates free radicals that initiate a lipid peroxidation reaction cascade [29]. ROS stimulate the release of pro-inflammatory mediators, leading to changes that generate more free radicals in a vicious cycle [29]. Ozone depletes levels of antioxidants in the stratum corneum and, thus, damages barrier function, also causing a series of cellular stress responses in the deeper layers of the skin [15]. This results in the production of 4-HNE, a marker of oxidative stress and lipid peroxidation that forms protein adducts associated with a variety of negative consequences to skin [30–32]. As reported in this study, pretreatment with WEL-DS significantly inhibited the formation of 4-HNE protein adducts following O_3_ exposure.

Through complex biological processes, oxidative stress leads to transient or permanent damage and activation of redox-sensitive transcription factors and signaling pathways involved in cell growth and differentiation as well as degradation of connective dermal tissue [29]. One of the most well studied of these is NF-κB. Activation of NF-κB involves the dissociation of the cytosolic NF-κB/IκB complex, which allows NF-κB to translocate into the nucleus, where it binds to its DNA recognition sequence and initiates gene transcription for several mediators such as growth factors and pro-inflammatory cytokines. Pretreatment with WEL-DS prevented the increase in NF-κB protein expression following O_3_ exposure; this may be an indicator of WEL-DS’s ability to inhibit O_3_-induced pro-inflammatory responses. Further investigation is needed to address this hypothesis.

Early research demonstrated that increasing exposure to O_3_ depletes levels of vitamins E and C in the skin [33]. More recent studies have shown that continued exposure to O_3_ is associated with an increased risk of skin conditions such as urticaria, eczema, and contact dermatitis [34–36]. Ground level O_3_ varies by location and other environmental pollutants; the concentration can range from 0.2 to 1.2 ppm in urban environments [37]. In this study, skin was exposed to O_3_ at 0.4 ppm. Ozone at 0.5 ppm is the level at which Los Angeles declares a Smog Alert No. 1. While sunscreens afford substantial benefit in protecting skin against UVA and UVB radiation, sunscreens alone offer no protection against the effects of O_3_ and other environmental elements. Sunscreens scatter, absorb, or block UV radiation prior to the formation of free radicals in the skin; topical antioxidants can penetrate the skin providing deeper protection [38]. Antioxidants neutralize free radicals and inhibit their capacity to inflict cellular damage. Exposure to O_3_ depletes antioxidants (such as vitamin E) in the skin; topical application of antioxidants can replenish antioxidant levels in the skin and protect the skin from O_3_-induced damage [13, 14, 33, 38]. Consequently, topical antioxidants assume a complementary role to sunscreens in providing optimal and comprehensive skin protection [17, 19, 23].

Previous research performed in a reconstructed human epidermal model investigated the ability of antioxidant mixtures containing vitamins C and/or E in preventing the deleterious effects of O_3_ on skin. Results demonstrated that pretreatment with the antioxidant mixtures reduced O_3_-induced oxidative stress [31].

Prior studies have demonstrated the benefits of WEL-DS in protecting skin against damage from UVA and UVB radiation [27]. An initial study evaluated the antioxidant capacity of WEL-DS in comparison to a leading antioxidant serum (L-AOX) and saline in human skin explants exposed to oxidative stress in two independent tests. A standardized hydrogen peroxide assay kit was used to detect peroxide activity in washed, homogenized skin tissue (20 µL of 0.1 mM hydrogen peroxide was used for each graft). Relative reduction in oxidative stress versus control was compared between WEL-DS and L-AOX. WEL-DS and L-AOX demonstrated significant antioxidant capacity in neutralizing hydrogen peroxide in human skin compared to saline; notably, skin treated with WEL-DS neutralized 53% and 41% increases in oxidative stress for L-AOX (test 1 and test 2, respectively). When tests were combined, mean oxidative stress over time was 33% significantly greater for L-AOX compared to WEL-DS (*p* = 0.012), indicating WEL-DS’s ability to quench hydrogen peroxide in comparison to saline and a leading antioxidant serum.

A second study assessed the ability of WEL-DS to protect skin against the oxidizing effects of UV radiation compared to an untreated, irradiated site in healthy women [27]. Compared to untreated, irradiated skin, pretreatment with WEL-DS led to significant reductions in UV-induced erythema at all minimal erythema dose (MED) exposures (1× MED, *p* = 0.025; 2× MED, *p* < 0.001; 3× MED, *p* = 0.004). Skin treated with WEL-DS also demonstrated significant reductions in thymine dimer formation (*p* < 0.02) and the upregulation of MMP-9 (*p* < 0.005). Skin pretreated with WEL-DS revealed substantial reductions in UV-stimulated sunburn cells and p53, in addition to significant protective effects to Langerhans Cells (*p* < 0.008) versus untreated skin.

A clinical trial evaluated the effectiveness and tolerability of WEL-DS for improvement in the appearance of facial aging caused by environmental or photodamage [27]. Twenty-two female subjects with mild to moderate facial photoaging were enrolled in this 12-week clinical study; 14 subjects were evaluated through 16 weeks. WEL-DS demonstrated visible improvements from baseline for fine lines/wrinkles (37%), erythema (18%), skin tone (17%), dyschromia (13%), and pore size (4%), with continued, progressive improvements achieved in all categories from baseline through week 16 in a subset of subjects. WEL-DS was well tolerated throughout the duration of the study.

Results from the present study provide additional support regarding the protective benefits of combinations of topical antioxidants against O_3_-induced damage to the skin. In the current study, pretreatment with a topical antioxidant serum containing a balanced ratio of 19 antioxidants designed to provide broad-based protection against free radical damage to skin significantly inhibited production of ROS, prevented the formation of hydrogen peroxide, and inhibited the formation of 4-HNE protein adducts in ozone-exposed tissues. Pretreatment with WEL-DS also inhibited structural damage and prevented the induction of NF-κB p65 protein in skin model tissues exposed to O_3_. Together, these findings demonstrate that WEL-DS is able to neutralize the effects of oxidative stress caused by O_3_ exposure.

In summary, application of a comprehensive topical antioxidant serum containing water-soluble, enzymatic, and lipid-soluble antioxidants, significantly diminished the effects of O_3_-induced oxidative damage in a reconstructed human epidermal skin model, suggesting that WEL-DS exhibits protective effects against daily O_3_ pollutant exposure.

## Abbreviations

O_3_: Tropospheric ozone
ROS: Reactive oxygen species
H_2_O_2_: Hydrogen peroxide
4-HNE: 4-Hydroxynonenal
NF-κB: Nuclear factor kappa-light chain enhancer of activated B cells
UV: Ultraviolet
MMP: Metalloproteinase
CO_2_: Carbon dioxide
Ppm: Parts per million
DCF-DA: Dichlorodihydrofluorescein diacetate
HRP: Horseradish peroxidase
NBF: Neutral-buffered formalin
L-AOX: Leading antioxidant
MED: Minimal erythema dose
